# Biomechanical and physiological effects of an upper-body exoskeleton during simulated load-carrying on an inclined surface

**DOI:** 10.1371/journal.pone.0325230

**Published:** 2025-06-03

**Authors:** Gabriela Garcia, Rafaella Yañez, Milena Espoz, Camilo Albuja, Paul G. Arauz, Bernard J. Martin

**Affiliations:** 1 Departamento de Ingeniería Industrial, Colegio de Ciencias e Ingenierías, Universidad San Francisco de Quito USFQ, Quito, Ecuador; 2 Department of Orthopaedics, Renaissance School of Medicine, Stony Brook University, New York, United States of America; 3 Department of Industrial and Operations Engineering, University of Michigan, Michigan, United States of America; Indian Institute of Technology Patna, INDIA

## Abstract

**Objective:**

This study assessed the effects of a passive upper-body exoskeleton (CarrySuit^®^) on heart rate, muscle activity, and kinematics while carrying 12 kg box on a 12° inclined treadmill.

**Background:**

Various passive exoskeletons designed for commercial use have emerged on the market, aiming to support lifting and carrying tasks. However, their effects on biomechanical metrics while walking on inclined surfaces are not yet conclusive.

**Method:**

Thirty participants carried a 12 kg box with and without the exoskeleton while walking on a treadmill with a 12° incline. Whole-body kinematics, heart rate, and muscle activity in the low back, legs, and arms were evaluated in each condition.

**Results:**

The exoskeleton significantly (p < .05) reduced peak erector spinae, biceps brachii activity, and heart rate across sexes, with medium to large effect sizes (η_p_^2 ^> 0.1). A decrease in mean erector spinae activity was observed in males only. However, for all, the exoskeleton increased vastus lateralis activity while reducing gastrocnemius activity, with medium effect sizes (η_p_^2 ^= 0.1). Kinematically, it led to increased dorsiflexion and knee flexion, with sex-specific adaptations such as reduced pelvic tilt in males and greater thorax tilt in females, with small to medium effect sizes. It also promoted a more neutral neck posture and altered hip asymmetry patterns.

**Conclusion:**

These findings suggest that the CarrySuit^®^ effectively alters heart rate, muscle activity and joint movements during inclined load-carrying tasks, with more benefits shown for males than females. This research contributes to the scientific understanding of commercial exoskeleton technology’s efficacy in carrying tasks.

## Introduction

Manual material handling tasks remain prevalent across diverse occupational sectors despite current trends in automation and mechanization. According to a recent survey, over 30% of workers frequently carry and move heavy loads [1]. Carrying and walking with heavy objects have been associated with an increased risk of musculoskeletal disorders (MSDs), notably affecting the lumbar region [2,3], the lower extremities [4], and the upper limbs [5,6]. Work-related musculoskeletal disorders continue to be a significant concern in various industries due to their substantial impact on worker well-being, productivity, and economic outcomes [7–10]. These disorders underscore the necessity for innovative interventions, as conventional strategies, such as automation or workplace modifications are not always feasible due to economic constraints or job characteristics [11–13]. Regarding the latter, numerous work environments require workers to handle heavy objects manually in areas where mechanical aids are impractical, such as staircases, uneven surfaces, or inclines, further exacerbating the risk of injury and emphasizing the importance of tailored ergonomic solutions.

In response to these challenges, recent technological advancements have facilitated the potential utilization of exoskeletons to mitigate physical strain and other factors associated with work-related musculoskeletal injuries [11–14]. Exoskeletons are wearable mechanical structures commonly categorized as active or passive. While active exoskeletons incorporate actuators and motorized components to augment human strength, passive exoskeletons utilize energy-storing materials such as springs or body-contact distribution to provide support during motion. The latter are generally less costly and commercially available for occupational use [15]. The majority of passive exoskeletons have been designed to support lifting tasks, prolonged static postures, and upper-limb work (see for review [11,12,16,17]) or walking without loads [18,19]. However, only a limited number have been designed and objectively evaluated during carrying and walking with heavy loads [20–22].

This study focuses on exploring the effects of exoskeletons on carrying and walking with a 12 kg box, utilizing the CarrySuit^®^, a passive upper-body exoskeleton developed by Auxivo AG (Auxivo AG, Schwerzenbach, Switzerland, https://www.auxivo.com/carrysuit). Unlike numerous exoskeletons designed to aid in bending and lifting, which have been the primary focus of research in recent years (as reviewed by de Looze et al. [13]), the CarrySuit^®^ is specifically designed to assist users in carrying, moving, and holding heavy objects; tasks commonly required in manual material handling jobs. Although few studies have examined their impact on walking while carrying heavy objects, these have primarily focused on subjective measures of discomfort and performance [23,24] with less emphasis on biomechanical outcomes [12,25]. Additionally, previous research on the CarrySuit^®^ [20,21] has exclusively evaluated carrying tasks on level surfaces. Therefore, the present study aims to uncover the influence of the exoskeleton in a more realistic scenario, including an inclined surface that simulates more extreme conditions that could occur in certain work environments, such as construction sites, steep outdoor terrains, or certain industrial settings where carrying could be particularly strenuous. For example, workers may need to carry heavy materials such as bricks, windows or tools up ramps during multi-story construction, transport supplies across uneven farmland, or move equipment in mining environments with sloped pathways. In these conditions, kinematics [26,27], postural stability [28,29], and muscular activity [30–32] can be affected.

Biomechanical and physiological aspects were evaluated through whole-body kinematics, muscular activity, and heart rate while carrying a heavy load on an inclined surface with and without the exoskeleton. These parameters were selected for their relevance in assessing physical demand and movements adaptations during manual material handling tasks. While our study did not directly assess postural stability (e.g., center or pressure or balance control metrics), whole-body kinematics and muscular activity offer valuable insight into the biomechanical impact of the exoskeleton, particularly in relation to posture maintenance and load distribution [11,33,34]. Impairments in these aspects can lead to compensatory and/or asymmetrical movements which may impose uneven loads on structures such as the lumbar spine [35]. This biomechanical imbalance can contribute to the development or exacerbation of MSDs, particularly under strenuous conditions, such as carrying a heavy load on an inclined surface. Therefore, investigating asymmetries in joint angles and muscle activity is critical to understanding their role in load distribution and potential injury risks [36]. Furthermore, comparing male and female participants is essential, as sex differences in perceived barriers to exoskeletons use have been previously noted [37]. Potential sex differences in physiological and biomechanical responses to exoskeleton use may influence the effectiveness and ergonomic benefits of the device, ultimately impacting injury prevention strategies in diverse working populations. Thus, the following research hypothesis were used in this study:

- Carrying a 12 kg load with the CarrySuit® on an inclined surface will significantly reduce heart rate and erector spinae and biceps brachii activity but increase the mean and peak muscular activity of the vastus lateralis and gastrocnemius medialis compared to the condition without the exoskeleton.
- Carrying a 12 kg load with the CarrySuit^®^ on an inclined surface will significantly influence whole-body sagittal plane joint angles, including the neck, thorax, pelvis, hip, knee, ankle, shoulder, and elbow angles, compared to the condition without the exoskeleton.
- There will be no significant difference between the left and right body segments (side) in joint angles and muscle activity, and any asymmetry observed will be consistent across conditions.
- Sex differences will not significantly influence overall outcomes.

## Methods

### Participants

Thirty young adults (15 females and 15 males) with no prior exposure to exoskeletons participated in this study as volunteers (see Table 1). Participants were recruited through university-wide announcements and social media posts. The final sample provided an achieved statistical power of 91%, based on a partial eta-squared (η_p_^2^) effect size of 0.1, and an alpha level of 0.05 (calculated using G*Power 3.1.9.7). Health status was evaluated through a pre-screening oral questionnaire completed prior to participation. Participants self-reported the absence of any current or past musculoskeletal disorders (e.g., chronic back pain, joint injuries, or repetitive strain conditions), pregnancy, or any physical or neurological conditions that could interfere with carrying a 12 kg load or wearing the passive exoskeleton used in this study.

**Table 1:** Participants demographic data.

	Males		Females	
	Mean	SD	Mean	SD
**Weight (kg)**	66.70	7,28	59.56	8.39
**Stature (cm)**	176.20	6,99	160.80	4.17
**Age (years)**	21.33	1.35	21.47	1.25

All participants were right-handed. The Ethics Committee of the Universidad San Francisco de Quito approved the study (#2021-145M), and the research adhered to the principles outlined in the Declaration of Helsinki. Written informed consent was obtained from each participant before engaging in the experimental sessions. Participants were recruited from until August 15, 2022 until December 31, 2022.

### Procedure

Upon arrival for the unique experimental session, participants were presented with the study details, and the use of the exoskeleton was demonstrated. Informed consent was obtained prior to data collection. Demographic information including age, sex, and handedness was self-reported, while stature and body mass were measured using a stadiometer and calibrated digital scale, respectively. Subsequently, the resting heart rate was determined. EMG electrodes were placed on the selected muscles, and respective isometric exercises for EMG data normalization were performed with three repetitions of 10-second, each separated by 1-minute rest periods. The isometric exercises consisted of the following submaximal voluntary contractions: 1) erector spinae: an extension of the trunk against a 5 kg resistance in the horizontal position, the Biering-Sorensen maneuver, [38]; 2) vastus lateralis: a knee extension and leg raised with 2.5 kg ankle weight [39]; 3) gastrocnemius medialis: while standing upright, a plantar-flexion holding a 7.5 kg kettlebell [40]; and 4) biceps brachii: an elbow flexion with a 7.5 kg kettlebell [41].

Markers were placed on the participants’ anatomical landmarks. A two-minute familiarization period was provided for participants to walk on the inclined treadmill while wearing the exoskeleton, allowing them to adjust to the walking speed before the test session. This duration was based on our previous study [42], where elevated muscle activation during the first two minutes of walking with the exoskeleton was observed. This result is also consistent with an initial adaptation period described in the literature [43], which likely reflects the time required for users to perceive the exoskeleton’s support and adapt their movement strategies accordingly. The baseline treadmill speed was determined based on each participant’s height and sex, following the methods-time measurement (MTM) approach [44], which is commonly used in time-motion studies to estimate efficient work rates for manual tasks. Although the MTM method does not directly account for load carriage, the speed was adjusted for each participant’s comfort and safety, while using the exoskeleton, to ensure realistic walking conditions while carrying a 12 kg load. Importantly, treadmill speed was fixed across conditions for each participant, including when wearing the exoskeleton, to control for variability in gait patterns and minimize confounding factors in biomechanical and physiological measurements.

A 12 kg box [41(L)x31(W)x28(H) cm] was carried at hip level with and without exoskeleton in a randomized order. This weight was chosen following the maximum acceptable weight for females according to Snook & Ciriello’s guidelines considering they needed to carry without aid over for one minute [45]. The carrying task was performed on a 12° inclined treadmill for one minute in each condition, with a 10-minute rest period in between. The incline was selected because it represents the maximum inclination of the treadmill to simulate more extreme conditions that could occur in certain work environments. Heart rate was recorded continuously during the task, with a sampling frequency of 1000 Hz. EMG and kinematic recordings were sampled at the 5^th^, 25^th^ and 45^th^ second for 15 seconds in each trial. This sampling strategy was designed to obtain representative gait cycles throughout the duration of the walking task, while minimizing the influence of potential adaptation at the initiation of movement, and avoiding data overload. A total of nine complete gait cycles for each limb were obtained and match for symmetry analysis. This study is part of a larger investigation in which participants also evaluated the exoskeleton during stair ascent and descent on the same experimental day, with a 30-minute break in between. The data from the stair analysis are presented elsewhere.

### Auxivo passive exoskeleton

**Fig 1:**
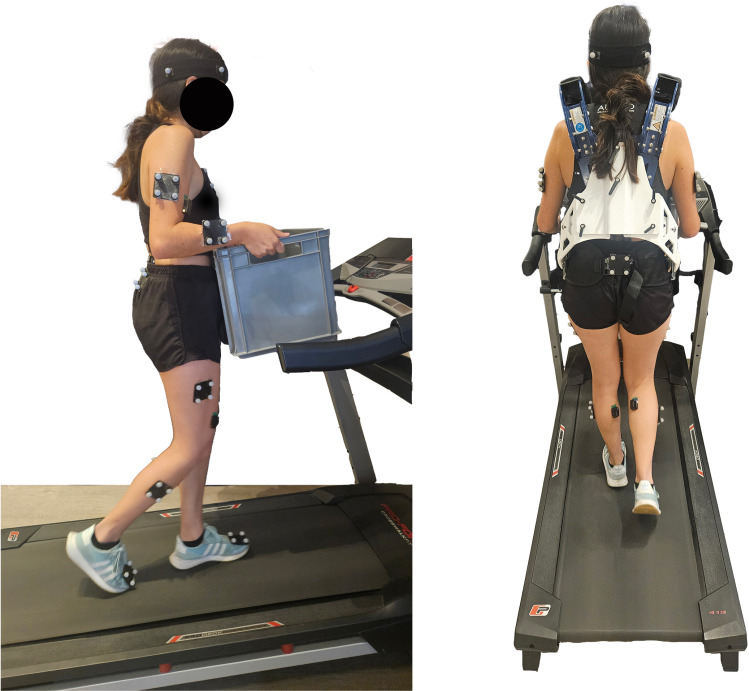
Participant wearing the CarrySuit^Ⓡ^ exoskeleton and walking on a treadmill with the highest inclination.

The carrying task was assessed with and without the passive upper-body exoskeleton Auxivo CarrySuit^Ⓡ^ v1.0 (Auxivo AG, Schwerzenbach, Switzerland). The CarrySuit^Ⓡ^ is designed to reduce strain on the upper extremities and dorsal region during the manual transportation of heavy objects. The exoskeleton weighs 5.6 kg and is engineered to support a maximum payload of 50 kg. It features a rigid, adjustable frame that spans from the hips to the shoulders, allowing adjustments to trunk length and hip width to accommodate different body sizes (Fig 1). The device is worn like a backpack, with textile straps and cushioned contact points connecting the hips, chest, and shoulders. Heavy objects are attached to the exoskeleton’s front using straps equipped with carabiners, which secure the load to the frontal frame. Load transfer is distributed mainly through the hips and shoulders, with no active energy storage (e.g., springs), meaning it provides support primarily during static carrying tasks, not during lifting or lowering movements.

### Measures and instrumentation

#### EMG.

Muscle activity was recorded bilaterally from the biceps brachii, erector spinae, vastus lateralis, and gastrocnemius medialis using wireless bipolar surface EMG sensors from Delsys Inc. (Boston, MA), including two sensors models: Trigno (earlier generation) and Avanti (newer generation). The activity of both biceps brachii was sampled at a rate of 1926 Hz using a pair of Trigno EMG sensors. The activity of the low back and leg muscles was sampled at 2148 Hz with Avanti EMGs sensors. The small difference in the fixed default sampling rates does not affect the validity of the data analyses.

Prior to sensor placement, the skin was prepared by shaving and applying abrasive gel (Skin Prep Gel, Nuprep®, Aurora, USA) to ensure optimal contact and low impendence. Sensor positioning adhered to SENIAM recommendations for each muscle (seniam.org) or previous research [46]. Vastus lateralis sensors were placed at 2/3 on the line from the anterior spina iliaca superior to the lateral side of the patella. Gastrocnemius medials sensors were placed on the most prominent belly of the muscle. Biceps brachii sensors were placed on the line between the medial acromion and the fossa cubit at 1/3 from the fossa cubit. Erector spinae sensors were placed at the 3^rd^ lumbar vertebra level, approximately 3 cm left and right from the spine [46].

Data were processed in MATLAB (MathWorks, Inc., Natick, MA), including detrending, filtering through a 4th-order Butterworth bandpass filter (30–300 Hz) [47,48], and Fast Fourier Transform conversion for data quality assessment. Signal quality was visually inspected by examining the power spectra to ensure the absence of abnormal peaks or irregularities indicative of noise or artifacts. Root mean square (RMS) values were calculated using a 250 ms moving window with 50% overlap. These RMS values were then normalized to a submaximal voluntary contraction, a widely accepted method [49,50], as described in the procedure section, for each muscle (biceps brachii, erector spinae, vastus lateralis, and gastrocnemius medialis). This allowed to express the results as percentages of the reference contraction. The primary outcome variables were the mean and peak normalized EMG amplitudes (RMS values) for each muscle. The mean normalized EMG amplitude represents the average muscle activation over the task duration, while the peak normalized EMG amplitude was defined as the 90th percentile of the normalized EMG values, capturing the highest sustained activation level during the task [51,52].

#### Kinematics.

**Fig 2:**
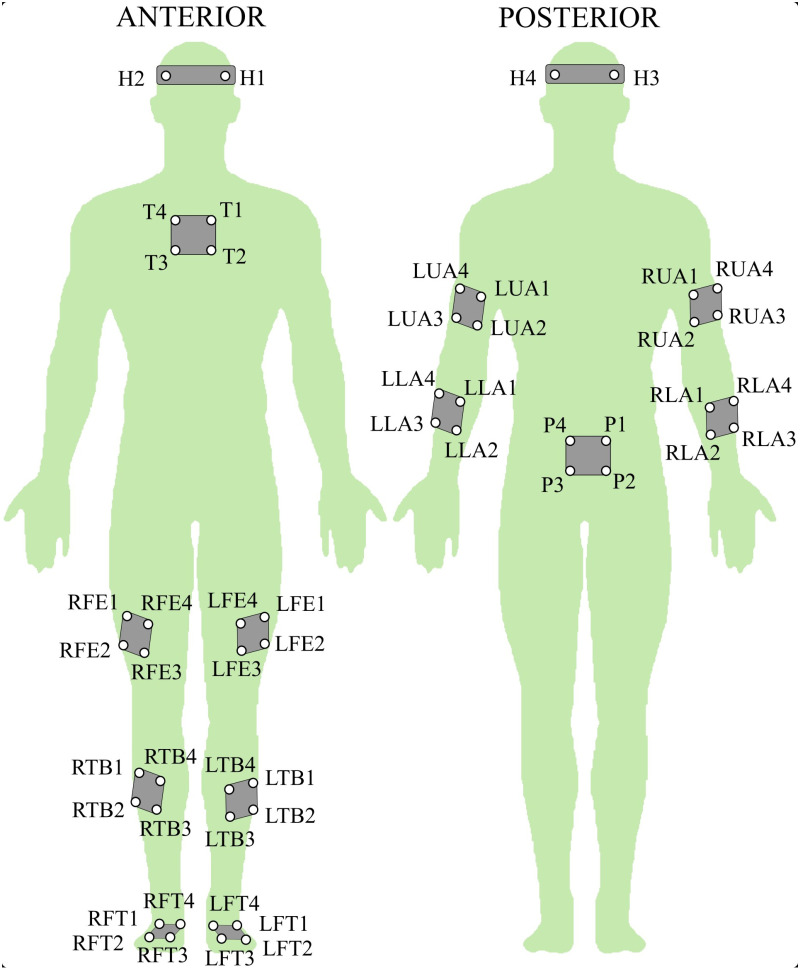
Whole-body marker set. Prefixes denote the following: L: Left, and R: Right presented before FE: femur, TB: tibia, FT: foot, LA: lower arm, UA, upper arm. Additionally, H: head, T: thorax, P: pelvis.

Whole-body kinematic data were acquired using a 10-camera motion capture system (Vicon MX, Oxford, UK) with a sampling frequency of 100 Hz. Clusters of four reflective spherical markers (10 mm in diameter) were meticulously attached to body segment landmarks, such as the head, thorax, pelvis, humerus, radius, femur, tibia, and foot, as depicted in Fig 2, to define their local coordinate systems. Building upon prior studies [53,54], a marker model consisting of 52 of these markers was employed to derive comprehensive whole-body kinematic information. Joint angles were calculated for the head-trunk, pelvis-trunk, shoulder (humerus-thorax), elbow (humerus-radius), hip (pelvis-femur), knee (femur-tibia), and ankle (foot-tibia) relative to a neutral standing posture, which was used as the zero reference. To extract joint angles, a Cardan angle sequence [55] was applied to compute flexion-extension, abduction-adduction, and axial rotation for each joint, though only the sagittal (flexion-extension) angles were selected for this study.

Motion data were recorded for 1-minute trials, both with and without the exoskeleton, and three 15-second segments from these trials were chosen for kinematic analysis. This choice ensured a representative sample of gait cycles, while maintaining manageable data processing. Strides were identified using heel contact events from the motion capture data, and the angular data were time-normalized to 100% of the gait cycle for comparability across participants. Each stride was defined from heel strike (0%) to the next heel strike (100%) of the same foot. Three complete gait cycles per limb were averaged for each condition [53,54,56].

The gait cycle was divided into five phases (0%, 20%, 40%, 60%, and 80%) to capture key moments of the walking motion and to potentially reveal subtle differences that may not be observed using traditional discrete parameters such as range of motion (ROM) [57,58]. These time points were chosen to represent critical biomechanical events such as heel strike, toe-off, and mid-swing, providing a simplified yet meaningful comparison of joint angles across conditions [59]. Statistical analysis focused on the sagittal plane joint angles at these five phases for both the upper and lower limbs, ensuring consistency in the evaluation of symmetry between the right and left sides. This segmentation allowed us to capture average joint angles at specific moments in the gait cycle, which enabled comparison of biomechanical patterns between the exoskeleton and non-exoskeleton conditions. Time-normalization ensured that variations in stride length and duration were controlled for, enhancing the reliability of our comparisons across participants.

#### Heart rate.

The heart rate in beats per minute (bpm) was assessed prior to and throughout the carrying task using a real-time Heart Rate Sensor System, Polar H10 (Polar ©, Finland), fastened to the chest. At the start of each experimental session, the lowest value of the resting heart rate was recorded while seated in an armchair for 5 minutes. Additionally, heart rate was tracked during the carrying task, and the average value was calculated.

### Data analysis

The statistical analyses were conducted in SAS Studio (SAS Institute Inc.) with a significance level α = .05. Mixed models were employed to analyze the outcome variables, including mean and peak muscular activity (derived from normalized EMG signals) and sagittal plane joint angles at each of the five phases of the gait cycle described above. These models incorporated a variance-components covariance structure with residual maximum likelihood estimation. All data fulfilled the normality tests.

In the analyses, participants were treated as random effects, while Sex (male or female), Condition (with exoskeleton [EXO] or without exoskeleton [NOEXO]), and Side (left or right body segments for symmetry analysis) were considered fixed effects. The model was simplified by excluding non-relevant interactions, specifically focusing on interactions with the condition factor, as the primary research interest was to assess the effect of the exoskeleton and its interaction with sex and side. Interactions such as Sex*Side were excluded because they were not hypothesized to influence the outcomes and did not significantly contribute to model fit. For the thorax, pelvis, and neck angles, as well as heart rate, the model included only the condition and sex factors. Post-hoc analyses were performed using least square means, and p-values were adjusted with the Tukey-Kramer method for accuracy. Post-hoc results are presented only if differences between conditions were found. Descriptive data are presented as mean ± standard error (SE). The partial eta-squared effect size was determined using the Tippey & Longnecker method for mixed models in SAS Studio [60]. Cohen’s benchmarks, which classify effect sizes as small (η_p_^2^ = .01), medium (η_p_^2^ = .06), and large (η_p_^2^ = .14), were applied to interpret the η_p_^2^ values [61].

## Results

### EMG

Statistical results for mean and peak muscular activity of the biceps brachii, erector spinae, vastus lateralis, and gastrocnemius medialis are presented in Table 2. The two-way interaction between Sex and Condition was significant only for the mean and peak biceps brachii and mean erector spinae activity, while the main effect of Condition was significant for all outcome variables with medium to large effect sizes, except for the peak activity of the gastrocnemius medialis. No significant two-way interaction between Side and Condition were found.

**Table 2:** Mean and peak normalized EMG amplitude mixed effects models results.

	p-values(η_p_^2^)							
	Biceps Brachii		Erector Spinae		Vastus Lateralis		Gastrocnemius Medialis	
Effect	Mean	Peak	Mean	Peak	Mean	Peak	Mean	Peak
Sex	.83(.0001)	.83(.0001)	.16(.01)	.09(.01)	.12(.01)	.23(.004)	.03*(.01)	.13(.01)
Condition	**<.0001** ^*^ **(.65)**	**<.0001** ^*^ **(.62)**	**<.0001** ^*^ **(.1)**	**.001** ^*^ **(.1)**	**<.0001** ^*^ **(.1)**	**<.0001** ^*^ **(.1)**	**.004** ^*^ **(.1)**	.28(.003)
Side	**.004** ^*^ **(.02)**	**.002** ^*^ **(.02)**	.38(.002)	.42(.001)	0.18(.01)	.07(.01)	**<.0001** ^*^ **(.1)**	**<.0001** ^*^ **(.1)**
Condition x Side	.39(.002)	.44(.001)	.08(.01)	.55(.001)	.72(.001)	.41(.001)	.94(.0001)	.62(.001)
Sex x Condition	**.03** ^*^ **(.01)**	**.01** ^*^ **(.02)**	**.002** ^*^ **(.03)**	.08(.01)	.27(.003)	.94(.00001)	.32(.002)	.55(.001)
Sex x Condition x Side	.11(.01)	.08(.01)	.05(.02)	.25(.01)	.31(.02)	.07(.01)	.56(.01)	.38(.01)

Note. Bold font and

*indicate significant values, α =.05.

Post-hoc analysis of the significant interactions and/or main effect of Condition, along with mean values and standard errors, are presented in Table 3. For the biceps brachii, both mean and peak muscular activity were significantly lower in the EXO condition compared to the NOEXO condition for both males and females. For the erector spinae, the mean muscular activity was significantly lower in the EXO condition than in the NOEXO condition for males, while no significant difference was found for females. However, peak activation was significantly lower in the EXO condition compared to the NOEXO condition, regardless of sex. For the vastus lateralis, both mean and peak muscular activity were significantly higher in the EXO condition than in the NOEXO condition, regardless of sex. For the gastrocnemius medialis, the mean muscular activity was significantly lower in the EXO condition than in the NOEXO condition, regardless of sex, with no significant difference found for peak activity.

**Table 3:** Mean and standard error for the biceps brachii, erector spinae, vastus lateralis, and gastrocnemius medialis mean and peak normalized EMG amplitude that presented significant interactions and/or main effect of condition.

		Normalized EMG Amplitude Mean(SE)%						
	Mean				Peak			
Muscle		EXO	NEXO	p-value		EXO	NEXO	p-value
Biceps Brachii	Males	30.05(7.53)	116.14(7.52)	<.0001*	Males	39.62(9.5)	144.34(9.5)	<.0001*
	Females	38.89(7.53)	111.58(7.54)	<.0001*	Females	52.51(9.51)	136.78(9.53)	<.0001*
Erector Spinae	Males	39.35(4.72)	47.87(4.73)	<.0001*	All	69.05(5.21)	73.99(5.22)	.001*
	Females	52.01(4.73)	54.01(4.74)	0.54				
Vastus Lateralis	All	46.17(3.60)	41.51(3.59)	<.0001*	All	107.15(9.01)	87.27(8.99)	<.0001*
Gastrocnemius Medialis	All	79.72(3.95)	84.76(3.94)	.004*	All	211.95(11.04)	216.82(11.03)	.28

Note. Values are presented as mean(standard error) in percentage of the reference contraction

### Kinematics upper and lower limbs

The mixed model analysis of sagittal plane angles obtained for the five phases of the gait cycle is presented in Table 4 for the upper and lower limbs. Overall, no significant three-way interaction was observed for these joint angles. A significant interaction of Condition and Side was found for the hips and elbows, while a significant interaction of Condition and Sex was found for the ankles, knees, hips, and shoulders. A significant main effect of Condition was found for most phases of the gait cycle for the knees, shoulders, and elbows. Post-hoc comparisons are summarized below for the significant two-way interactions of the evaluated joint angles.

**Table 4:** Statistical fixed effects results for sagittal plane joint angles of the ankles, knees, hips, shoulders, and elbows at different phases of the gait cycle.

Joint	Effect														
	p-values (η_p_^2^)														
	Sex					Condition					Side				
	0%	20%	40%	60%	80%	0%	20%	40%	60%	80%	0%	20%	40%	60%	80%
Ankles	.63(.001)	.89(.0001)	.67(.0005)	.23(.004)	.39(.002)	.88(.0001)	.41(.002)	.93(.00002)	.94(.00001)	**.001** ^*^ **(.03)**	.15(.006)	**.03** ^*^ **(.01)**	.92(.00002)	.83(.0001)	.06(.01)
Knees	.23(.004)	.98(.00001)	.43(.002)	.86(.0001)	.81(.0002)	**<.001** ^*^ **(.13)**	**<.001** ^*^ **(.04)**	.07(.01)	**.006** ^*^ **(.02)**	**<.001** ^*^ **(.07)**	**<.001** ^*^ **(.04)**	**<.001** ^*^ **(.04)**	.24(.004)	**.01** ^*^ **(.02)**	**.02** ^*^ **(.01)**
Hips	.1(.008)	.89(.0001)	.91(.00003)	.27(.003)	.88(.0001)	**<.001** ^*^ **(.11)**	.08(.01)	.97(.000002)	.13(.006)	.18(.005)	.11(.007)	.17(.005)	**<.001** ^*^ **(.09)**	**<.001** ^*^ **(.09)**	.46(.002)
Shoulders	.81(.0002)	.82(.0001)	.92(.00001)	.91(.00003)	.81(.0001)	**<.001** ^*^ **(.25)**	**<.001** ^*^ **(.25)**	**<.001** ^*^ **(.18)**	**<.001** ^*^ **(.2)**	**<.001** ^*^ **(.23)**	**.002** ^*^ **(.03)**	**.006** ^*^ **(.02)**	**.01** ^*^ **(.02)**	**.01** ^*^ **(.02)**	**.007** ^*^ **(.02)**
Elbows	.79(.0001)	.69(.0004)	.5(.001)	.56(.001)	.85(.0001)	**.001** ^*^ **(.02)**	**<.001** ^*^ **(.05)**	**<.001** ^*^ **(.04)**	.11(.007)	<.001*(.02)	**<.001** ^*^ **(.07)**	**<.001** ^*^ **(.13)**	**<.001** ^*^ **(.11)**	**<.001** ^*^ **(.07)**	**<.001** ^*^ **(.1)**
Joint	**Effect**														
	**p-values**														
	Sex*Condition					Condition*Side					Sex*Condition*Side				
	0%	20%	40%	60%	80%	0%	20%	40%	60%	80%	0%	20%	40%	60%	80%
Ankles	**<.001** ^*^ **(.04)**	**0001** ^*^ **(.03)**	.06(.01)	.21(.004)	.93(.00003)	.39(.004)	.46(.002)	.34(.003)	.51(.001)	.09(.008)	.81(.0002)	.85(.005)	.68(.003)	.76(.009)	.05(.03)
Knees	**<.001** ^*^ **(.06)**	.01*(.02)	.19(.004)	.53(.001)	.11(.008)	.76(.002)	.41(.002)	.26(.004)	.47(.002)	.57(.0009)	.21(.02)	.21(.007)	.42(.002)	.3(.04)	.17(.006)
Hips	.18(.005)	.12(.007)	.58(.0009)	.33(.003)	**.03** ^*^ **(.01)**	**.005** ^*^ **(.02)**	**<.001** ^*^ **(.04)**	**<.001** ^*^ **(.06)**	**<.001** ^*^ **(.04)**	.08(.008)	.32(.07)	.98(.03)	.33(.01)	.2(.03)	.26(.05)
Shoulders	**<.001** ^*^ **(.16)**	<.001*(.13)	**<.001** ^*^ **(.13)**	**<.001** ^*^ **(.13)**	**.001** ^*^ **(.14)**	.81(.0001)	.99(.00001)	.97(.00001)	.82(.0001)	.58(.0001)	.66(.003)	.92(.001)	.85(.003)	.85(.004)	.61(.01)
Elbows	.26(.003)	.29(.003)	.22(.004)	.31(.003)	.56(.001)	**<.001** ^*^ **(.04)**	**<.001** ^*^ **(.08)**	**<.001** ^*^ **(.07)**	**<.001** ^*^ **(.02)**	**<.001** ^*^ **(.06)**	.61(.02)	.48(.02)	.25(.02)	.33(.005)	.75(.03)

Note. Bold font and

*indicates significant values, α =.05.

**Fig 3:**
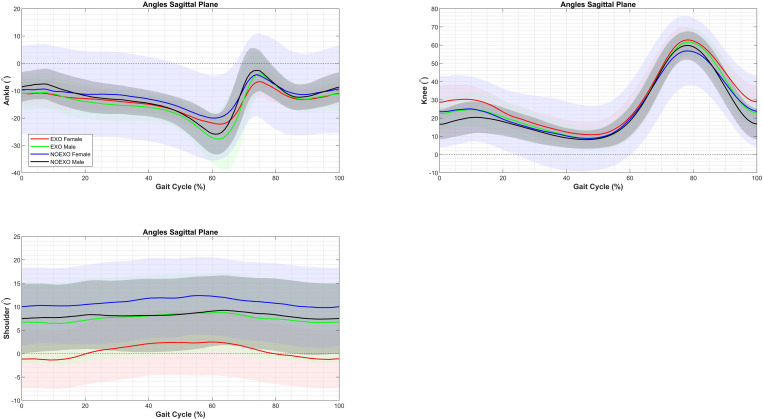
Average and standard deviation of ankles, knees, and shoulders joint angles in the sagittal plane for males and females both with (EXO) and without (NOEXO) the exoskeleton during a gait cycle of inclined treadmill walking. Solid lines represent average angles, while shaded areas denote standard deviations.

Differences between Conditions and Sex, regardless of Side, were observed for the ankles, knees, and shoulders joints, as shown in Fig 3. For males only, dorsiflexion was greater with the exoskeleton than without it at both the start 0% (EXO: −10.55 ± 1.01°; NOEXO: −8.93 ± 1.01°; *p* < .001) and 20% (EXO: −12.99 ± .99°; NOEXO: −11.44 ± .99°; *p* < .001) of the gait cycle. Similarly, only male participants showed increased knee flexion with the exoskeleton at the start 0% (EXO: 23.48 ± 1.94°; NOEXO: 17.48 ± 1.93°; *p* < .001), and 20% (EXO: 21.08 ± 1.49°; NOEXO: 18.69 ± 1.50°; *p* < .001) of the gait cycle. Conversely, for females only, shoulder flexion was consistently lower with the exoskeleton at all phases (*p* < .001) of the gait cycle (0%: EXO: −.68 ± 1.66°; NOEXO: 10.48 ± 1.65°; 20%: EXO:.45 ± 1.68°; NOEXO: 10.94 ± 1.68°; 40%: EXO: 2.25 ± 1.71°; NOEXO: 12.01 ± 1.70°; 60%: EXO: 2.75 ± 1.70°; NOEXO: 12.37 ± 1.69°; 80%: EXO: −.61 ± 1.68°; NOEXO: 11.08 ± 1.67°).

**Fig 4:**
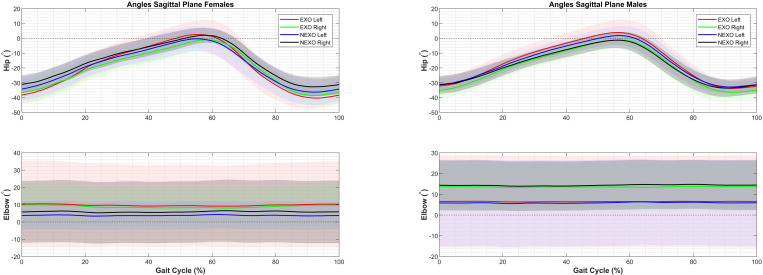
Average and standard deviation illustrate the side differences between the left and right hips, as well as elbows joint angles in the sagittal plane, both with (EXO) and without (NOEXO) the exoskeleton during a gait cycle of inclined treadmill walking. Solid lines represent average angles, while shaded areas denote standard deviations.

Differences between Conditions and Side, regardless of Sex, were observed for the hips and elbows, as shown in Fig 4. For the hips, asymmetry was present with the exoskeleton (EXO) at 0% (Left: −34.92 ± .85°; Right: −35.51 ± .86°; *p* = .04), 20% (Left: −18.41 ± .93°; Right: −20.85 ± .94°; *p* = .02), 40% (Left: −3.64 ± 1.01°; Right: −8.68 ± 1.01°; *p* < .001), and 60% (Left: 3.67 ± 1.12°; Right: −1.25 ± 1.13°; *p* < .001) of the gait cycle, where greater hip extension was observed on the right side. Without the exoskeleton, a side difference appeared only at 0% with greater hip extension on the left side (NOEXO: Left: −33.23 ± .86°; Right: −31.07 ± .86°; *p* < .001).

For the elbows, asymmetry was found only in the condition without the exoskeleton, with greater right elbow flexion at all phases (*p* < .001) of the gait cycle (NOEXO: 0%: Left: 1.28 ± 2.08°; Right: 13.36 ± 2.10°; 20%: Left: −3.89 ± 2.04°; Right: 13.15 ± 2.06°; 40%: Left: −2.83 ± 1.98°; Right: 13.06 ± 2.01°; 60%: Left: 1.92 ± 2.01°; Right: 13.79 ± 2.03°; 80%: Left: −.81 ± 2.02°; Right: 13.79 ± 2.04°).

### Kinematics thorax, pelvis and neck

The mixed model analysis results for sagittal plane angles of the pelvis, thorax, and neck across five phases of the gait cycle are presented in Table 5. A significant two-way interaction between Sex and Condition was found for the pelvis and thorax with small to medium effect sizes, while a main effect of Condition was observed for the neck with medium effect sizes. Post-hoc comparisons for these significant interactions and main effects are summarized below.

**Table 5:** Statistical fixed effects results for sagittal plane joint angles of the pelvis, thorax, and neck at different phases of the gait cycle.

Joint	Effect														
	p-values(η_p_^2^)														
	Sex					Condition					Sex*Condition				
	0%	20%	40%	60%	80%	0%	20%	40%	60%	80%	0%	20%	40%	60%	80%
Pelvis	.66(.001)	.77(.0001)	.83(.0003)	.76(.0001)	.83(.0002)	.24(.02)	.07(.02)	.02^*^(.03)	.11(.02)	.05(.03)	**.04** ^*^ **(.03)**	**.03** ^*^ **(.04)**	**.02** ^*^ **(.03)**	**.01** ^*^ **(.04)**	**.02** ^*^ **(.03)**
Thorax	.08(.01)	.06(.01)	.08(.01)	.08(.01)	.05(.01)	**<.001** ^*^ **(.06)**	**.005** ^*^ **(.04)**	**.01** ^*^ **(.05)**	**.006** ^*^ **(.06)**	**.002** ^*^ **(.06)**	**.002** ^*^ **(05)**	**.004** ^*^ **(.05)**	**.006** ^*^ **(.05)**	**.004** ^*^ **(.05)**	**.003** ^*^ **(.05)**
Neck	.17(.01)	.13(.01)	.21(.004)	.11(.01)	.18(.004)	**<.001** ^*^ **(.1)**	**.001** ^*^ **(.07)**	**.001** ^*^ **(.08)**	**<.001** ^*^ **(.1)**	**<.001** ^*^ **(.08)**	.19(.01)	.42(.01)	.52(.005)	.19(.01)	.21(.01)

Note. Bold font and

*indicates significant values, α =.05.

**Fig 5:**
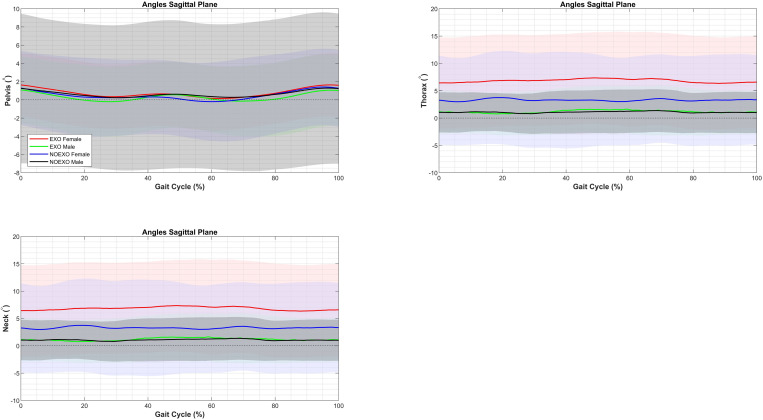
Average and standard deviation of pelvis, thorax, and neck sagittal plane angles for males and females both with (EXO) and without (NOEXO) the exoskeleton during a gait cycle of inclined treadmill walking. Solid lines represent average angles, while shaded areas denote standard deviations.

Differences between Condition and Sex were observed for the pelvis and thorax angles, as shown in Fig 5. For males, pelvis tilt was significantly lower with the exoskeleton than without it across all gait cycle phases (0%: EXO: −.45 ± .80°; NOEXO: 1.73 ± .80°; *p* = .04; 20%: EXO: −.23 ± .83°; NOEXO: −1.42 ± .83°; *p* = .01; 40%: EXO:.69 ± .81°; NOEXO: 2.48 ± .81°; *p* = .003; 60%: EXO:.42 ± .80°; NOEXO: 2.11 ± .81°; *p* = .001; 80%: EXO: −.34 ± .83°; NOEXO: 1.37 ± .84°; *p* = .01). In contrast, for females, thorax anterior tilt was significantly higher with the exoskeleton across all phases (0%: EXO: 6.49 ± 1.32°; NOEXO: 1.87 ± 1.32°; *p* < .001; 20%: EXO: 6.29 ± 1.29°; NOEXO: 2.32 ± 1.29°; *p* = .001; 40%: EXO: 5.41 ± 1.28°; NOEXO: 1.88 ± 1.29°; *p* = .003; 60%: EXO: 5.49 ± 1.25°; NOEXO: 1.79 ± 1.26°; *p* = .001; 80%: EXO: 6.01 ± 1.29°; NOEXO: 1.92 ± 1.30°; *p* < .001).

For the neck, only Condition differences were noted, with a consistently higher anterior tilt angle without the exoskeleton across all phases (*p* < .001) of the gait cycle (0%: EXO: −1.42 ± 1.73°; NOEXO: 3.61 ± 1.73°; 20%: EXO: −.66 ± 1.68°; NOEXO: 3.34 ± 1.68°; 40%: EXO: −.12 ± 1.69°; NOEXO: 4.24 ± 1.68°; 60%: EXO: −1.13 ± 1.62°; NOEXO: 3.75 ± 1.61°; 80%: EXO: −.13 ± 1.65°; NOEXO: 4.62 ± 1.64).

### Heart rate

The statistical analysis revealed a significant main effect of condition on heart rate (*p* < .0001; η_p_^2 ^= .25), with no significant effect of sex. Post-hoc comparisons indicated a significantly lower mean heart rate during the EXO condition (M = 111.50, SE = 3.45 bpm) compared to the NOEXO condition (M = 119.13, SE = 3.40 bpm). Resting heart rates before each experimental condition, regardless of sex, were not significantly different (EXO: M = 77.07, SE = 2.56 bpm; NOEXO: M = 75.61, SE = 2.57 bpm; *p* = 0.06) and were both significantly lower than the mean activity heart rates (*p* < .0001).

## Discussion

While walking on an inclined surface, the influence of the CarrySuit^®^, an upper-body exoskeleton designed to aid in carrying tasks, was evaluated through kinematics, muscle activity, and heart rate. Our results revealed that the peak EMG activity of the erector spinae muscles on each side required to carry a 12 kg load while walking on an inclined surface was lower with than without the exoskeleton for both males and females. A similar reduction was observed in two previous studies evaluating the CarrySuit^®^ during a carrying task while walking on a level surface [20] or on a horizontal treadmill [21]. Mean erector spinae activation was lower with the exoskeleton for males, while no significant changes were observed for females; similarly, studies on level-surface walking have reported no increase in lower back muscle activation with exoskeleton use [20,21]. Overall, the lower activation with the exoskeleton indicates that the added load from the CarrySuit’s weight is not supported by the lower back muscles, demonstrating a positive benefit of the exoskeleton for carrying load on inclined surfaces.

As expected, the lower arm muscle activity with the exoskeleton, shown by the reduction in biceps brachii mean and peak activations, corroborated our previous study [20]. For the legs, the increased peak activation of the vastus lateralis suggests that the exoskeleton shifted load support to the knee extensors muscles. However, this shift did not extend to the ankle plantarflexor muscles, as gastrocnemius mean activation was lower, and peak activation did not increase with the exoskeleton. Similar results were observed in Garcia et al. [22] for the CarrySuit^®^ on a level surface, while Riemer et al. [25], studying a back-support exoskeleton during load carrying/walking, found increased gastrocnemius activation. These differences may be specific to the exoskeleton designs.

With the exception of the vastus lateralis, muscle activation of the upper body was reduced with the exoskeleton, which aligns with a reduction in overall exertion, as indicated by the lower mean heart rate in this condition. Consistent with previous studies on carrying task, this indicator of cardiovascular effort was lower with the CarrySuit^®^ than without it [20,21].

Furthermore, this study revealed a greater dorsi and knee flexion in males at the beginning (0% and 20%) of the gait cycle with the exoskeleton. These changes were not observed in females. Similar changes in ankle and knee flexion were previously noted when carrying a load on a level surface with the CarrySuit^®^ [22], and with a similar back-support exoskeleton [25]. This may be because participants modified their initial posture due to the exoskeleton and added weight, which seems to transfer the load to the legs, consistent with the increased activity of the vastus lateralis. Additionally, previous studies of overground gait analysis have shown that males tend to exhibit higher knee flexion during the initial phase of the gait cycle [62,63]. Therefore, the lack of change in females is likely due to sex differences in postural behavior rather than the tested exoskeleton. Nevertheless, no differences were found in the middle of the gait cycle between the exoskeleton and no exoskeleton conditions for both sexes.

For the hips, both males and females showed greater right hip extension from the start to 40% of the gait cycle when using the exoskeleton. Similar increases in hip extension have been noted when carrying heavy backpacks while walking [64] and when carrying a box with a back-support exoskeleton [25], though these studies did not include side comparison. Without the exoskeleton, hip asymmetry was present only at the start of the gait cycle (0%). Previous studies report no hip asymmetry in healthy males and females during normal or fast overground walking [63,65], although significant asymmetry has been observed during normal and fast treadmill walking without incline [54]. Therefore, the pronounced asymmetry observed with the exoskeleton may reflect increased upper leg effort, as indicated by higher vastus lateralis activation discussed above, and the interaction with treadmill walking. This could relate to adaptation to both the exoskeleton and treadmill, as recent research suggest gait asymmetries can arise in response to specific task demands [66].

Asymmetry between the elbows was observed throughout all phases of the gait cycle, but only without the exoskeleton. In this condition, participants exhibited greater flexion in the right elbow than the left, likely due to laterality, as all participants were right-hand dominant [65]. When using the exoskeleton to support the load, the arms maintained a more relaxed posture, primarily involved in controlling lateral load movements. As a result, participants tended to rest their arms at the sides of the box with a more natural flexion, leading to no observed asymmetry with the exoskeleton.

Furthermore, the analysis of thorax, pelvis, and neck movements revealed distinct effects of exoskeleton usage and sex. In males, pelvic tilt decreased with the exoskeleton across all phases of the gait cycle, as the angles were in a more neutral position compared to carrying the load without the exoskeleton. Since the CarrySuit^®^ is fixed over the pelvis, its belt may help maintain the pelvis in a neutral position during movement. Similar changes at the pelvic level has been previously observed in gait studies with loaded backpacks on both level and inclined surfaces [67] and with the CarrySuit^®^ on level surfaces [22]. However, a sex difference was not previously noted.

Contrary to the pelvic results, anterior thorax tilt increased with the exoskeleton only in females. Our previous study showed a greater range of motion in the thorax only in females when using the exoskeleton on a level surface [22]. Increased trunk forward tilt has been previously observed during level and inclined walking with loaded backpacks, particularly during the stance phase, as reviewed by Genitrini et al. [67]. However, given that erector spinae mean activity was not affected by the exoskeleton in females, their increased thorax tilt may represent a compensatory postural strategy when using the device. In contrast, males seem to maintain a stiffer trunk posture when wearing the exoskeleton, with no changes observed across conditions for them.

For the neck, the position was closer to neutral with the exoskeleton and showed a higher anterior tilt without the exoskeleton for both males and females throughout all phases of the gait cycle. This difference suggests that users maintain a more relaxed neck posture during the carrying task when using the exoskeleton. This observation aligns with the lower middle trapezius muscle activity and the absence of neck discomfort reported in previous CarrySuit^®^ evaluations [21]. Overall, pelvic, and trunk postures in males, and neck posture in both males and females, tended to be more neutral with the CarrySuit^®^, contributing to a less physically demanding tasks. However, the increased trunk flexion observed in females is of concern and may warrant further investigation.

### Study limitations

The measurements were conducted within a controlled laboratory environment; thus, caution should be taken when generalizing the results to real-world manual material handling tasks. The outcome measures are limited to the effects of the tested exoskeleton, leaving the long-term consequences unexplored, especially during a full workday or extended periods of use. The study’s participant pool predominantly consisted of relatively young individuals, implying a potential difference of the exoskeleton’s impact on individuals of diverse age groups commonly found in the workplace. Despite a familiarization period provided before the experimental task, adaptation effects should be considered. The experimental condition only included a positive slope, descending on a ramp while carrying and different degrees of slopes should be considered in future studies. Moreover, the present study did not include lifting and lowering the load from the ground, which is commonly observed in industry. Also, it did not include a condition in which the exoskeleton was worn with the support mechanism deactivated. Including such a condition could help isolate the mechanical influence of the support system itself and better reflect real-world usage scenarios where users may deactivate support during certain tasks. Future research should explore this aspect to further evaluate the exoskeleton’s impact on movement and usability. Although subjective measures of perceived exertion and exoskeleton usability were collected as part of the larger study, these results are reported elsewhere and were not included in this manuscript. Future studies may explore how these subjective responses align with the objective biomechanical findings presented here.

## Conclusions

The present study evaluating the effects of passive exoskeleton CarrySuit^®^ on biomechanical and physiological parameters during inclined walking with a 12 kg load illustrates the potential benefits of using this device to carry heavy loads. The utilization of the exoskeleton consistently reduced biceps brachii and erector spinae muscle activity, as well as heart rate, indicating potential benefits in terms of muscular effort and cardiovascular demand. However, a sex-specific difference was found for erector spinae mean activity favoring males. Conversely, the vastus lateralis exhibited increased activity with the exoskeleton, while the gastrocnemius medialis showed decreased mean activity, suggesting a complex interplay between the device and specific muscle groups. In terms of kinematics, the device increased dorsiflexion, knee flexion, and reduced pelvic tilt in males, and increased anterior thorax tilt in females; however, for both sexes it encouraged a neutral neck posture. Overall, in males, the pelvis, trunk, and neck posture tend to be more neutral and less physically demanding when using the CarrySuit^®^, except for some asymmetries observed at the hips. These improvements are likely the result of a better distribution of forces despite the weight of the device. Furthermore, these findings also underscore the importance of considering sex differences and the specific conditions of exoskeleton use when evaluating their effectiveness and impact on gait and posture.

## Supporting information

S1 File PLOS One's inclusivity in global research questionnaire. (DOCX)
